# A Novel, Highly Related Jumbo Family of Bacteriophages That Were Isolated Against *Erwinia*

**DOI:** 10.3389/fmicb.2019.01533

**Published:** 2019-07-23

**Authors:** Ruchira Sharma, Brittany A. Pielstick, Kimberly A. Bell, Tanner B. Nieman, Olivia A. Stubbs, Edward L. Yeates, David A. Baltrus, Julianne H. Grose

**Affiliations:** ^1^Department of Microbiology and Molecular Biology, Brigham Young University, Provo, UT, United States; ^2^School of Plant Sciences, The University of Arizona, Tucson, AZ, United States

**Keywords:** jumbo bacteriophage, *Agrican357virus*, burst size, novel, *Erwinia*, *Pantoea*, genome, proteome

## Abstract

*Erwinia amylovora* is a plant pathogen from the *Erwiniaceae* family and a causative agent of the devastating agricultural disease fire blight. Here we characterize eight lytic bacteriophages of *E. amylovora* that we isolated from the Wasatch front (Utah, United States) that are highly similar to vB_EamM_Ea35-70 which was isolated in Ontario, Canada. With the genome size ranging from 271 to 275 kb, this is a novel jumbo family of bacteriophages. These jumbo bacteriophages were further characterized through genomic and proteomic comparison, mass spectrometry, host range and burst size. Their proteomes are highly unstudied, with over 200 putative proteins with no known homologs. The production of 27 of these putative proteins was confirmed by mass spectrometry analysis. These bacteriophages appear to be most similar to bacteriophages that infect *Pseudomonas* and *Ralstonia* rather than *Enterobacteriales* bacteria by protein similarity, however, we were only able to detect infection of *Erwinia* and the closely related strains of *Pantoea*.

## Introduction

Whitman et al. (1998) estimated that there are approximately 5 × 10^30^ bacteria on earth, which is more than the number of plants and animals combined. Most, or likely all, bacteria are subject to infection by one or more viruses or “bacteriophages,” making bacteriophages the most common and diverse biological entity at an estimated 10^32^ (Bergh et al., 1989; Wommack and Colwell, 2000; Hambly and Suttle, 2005). Bacteriophages were likely first reported in 1896, when Ernest Hanbury Hankin discovered antibacterial activity against cholera in the waters of two large rivers in India, the Ganges and Yamuna (Abedon et al., 2011). They were independently characterized and named in the 1900s by bacteriologist Twort (1915, 1936) and microbiologist D’Herelle (1917, 2007), Keen (2015). During the infection process, bacteriophages can transfer foreign DNA to their host (including virulence factors), integrate into the host genome, and/or kill their host through cell lysis (Chen and Novick, 2009). The sheer number of bacteriophages combined with their clear evolutionary influence makes them an important target for understanding the ecology and evolution of bacteria, including pathogenic strains (Bollback and Huelsenbeck, 2009; Boyd, 2012). In addition, their specificity, genomic plasticity, and rapid multiplication rates make them a potential weapon to treat bacterial infections (Sulakvelidze, 2005; Shivaswamy et al., 2015).

One such bacterial infection caused by a phytopathogen *Erwinia amylovora* (Zwet and Keil, 1979) is called fire blight and mainly affects ornamental plants of the *Rosaceae* family. The symptoms of the infected tissues include wilting, ooze production and death of blossoms, shoots branches and entire trees (Thomson, 2000). We have recently isolated and characterized twenty eight bacteriophages that infect *E. amylovora* (Esplin et al., 2017; Sharma et al., 2018). Out of these 28, there is a distinct group of eight highly related bacteriophages: vB_EamM_Special G (Special G), vB_EamM_Simmy50 (Simmy50), vB_EamM_RAY (RAY), vB_EamM_Deimos-Minion (Deimos-Minion or DM), vB_EamM_Bosolaphorus (Bosolaphorus), vB_EamM_Desertfox (Desertfox), vB_EamM_MadMel (MadMel), and vB_EamM_Mortimer (Mortimer) very similar to *Erwinia* bacteriophage Ea35-70 which was isolated in Ontario, Canada (Yagubi et al., 2014). These nine bacteriophages were recently added as the *Agrican357virus* genus of bacteriophages by the ICTV (Kuhn et al., 2013; Adams et al., 2017) and are considered jumbo bacteriophages due to their large genome (>200 kb) and particle size (Yuan and Gao, 2017).

As reviewed in 2017, jumbo bacteriophages have diverse genome sizes (ranging from 208 to 497 kb) as well as diverse virion morphology and complex virion structure (Yuan and Gao, 2017). They often encode greater than 60 structural proteins with some displaying complex head structures composed of more than five proteins (Effantin et al., 2013) or long, wavy, curly tail fibers (Yuan and Gao, 2016). Jumbo bacteriophages were also found to be highly diverse, with over 11 clusters and five singleton bacteriophages suggested from 52 complete jumbo bacteriophage genomes analyzed in 2017, many of which are uncharacterized (Yuan and Gao, 2017). Only a few jumbo bacteriophage families have been characterized beyond sequence analysis and EM, including the phiKZ-like bacteriophages 201phi2-1 (Thomas et al., 2008), KTN4 (Danis-Wlodarczyk et al., 2016), phiPA3 (Monson et al., 2011), phiRSL2 (Bhunchoth et al., 2016), phiRSF1 (Bhunchoth et al., 2016), OBP (Shaburova et al., 2006), EL (Sokolova et al., 2014), and phiKZ (Lecoutere et al., 2009), related bacteriophages phiRSL1 (Yamada et al., 2010) and PaBG (Kurochkina et al., 2018), *Cronobacter* bacteriophage CR5 (Lee et al., 2016), *Prochlorococcus* bacteriophage P-SSM2 (Sullivan et al., 2005), related bacteriophages KVP40 (Miller et al., 2003) and Aeh1 (Gibb and Edgell, 2007), *Aeromonas* bacteriophage phiAS5 (Kim et al., 2012), *Pectobacterium* bacteriophage CBB (Buttimer et al., 2017), *Caulobacter* bacteriophage phiCbK (Gill et al., 2012), related *Erwinia* bacteriophages Joad and RisingSun (Arens et al., 2018), related bacteriophage RaK2 (Simoliunas et al., 2013) and GAP32 (Abbasifar et al., 2014), *Bacillus* bacteriophage 0305phi8-36 (Thomas et al., 2007), related *Bacillus* bacteriophages BpSp (Yuan and Gao, 2016) and AR9 (Lavysh et al., 2016, 2017). Herein we further analyze the genome, proteome, and host range of our eight *Agrican357virus* jumbo bacteriophages. Their lytic nature and plethora of novel genes makes them a unique entity to be studied further and analyzed. As a close relative of the animal pathogens *Escherichia coli* and *Salmonella* (Zhao and Qi, 2011), viruses that infect *E. amylovora* may help us understand the evolution of pathogenic strains in this family.

## Materials and Methods

### Bacteriophage Isolation and Genome Sequencing

Environmental samples of leaves, branches and soil surrounding infected trees were collected from around the state of Utah (United States) and used to create enrichment cultures with the host *E. amylovora*. To test the presence of amplified bacteriophages, the enrichment cultures were spun at 4000 rpm and 4°C for 20 min and the supernatant was removed and used without filtering. 50 μL of this supernatant was incubated at room temperature with 500 μL of *E. amylovora* ATCC 29780 bacteria for 30–45 min, mixed with 5 ml NBDYE top agar (at half concentration agar), plated on NBSYE agar plate, and incubated at 25°C overnight. Plaque presence on the plates was the primary indicator of bacteriophage presence in the environmental sample. Using a sterile needle or pipette tip, we picked a plaque from the initial bacteriophage identification plate and performed three rounds of plaque purification. All eight isolated bacteriophages: Special G (KU886222), Simmy50 (KU886223), RAY (KU886224), Deimos-Minion (KU886225), Bosolaphorus (MG655267), Desertfox (MG655268), MadMel (MG655269), and Mortimer (MG655270) were able to infect *E. amylovora* ATCC 29780 (Esplin et al., 2017; Sharma et al., 2018). Bacteriophage DNA was extracted using the Phage DNA isolation kit (Norgen Biotek Corporation), and was sequenced, assembled and annotated as previously described (Esplin et al., 2017; Sharma et al., 2018).

### Electron Microscopy

Electron microscopy was performed at Brigham Young University in the Life Sciences Microscopy Lab using a FEI Helios NATOCAB 600i DualBeam FIB/SEM with STEM detector. The samples for SEM analysis were prepared by placing 15 μL of high-titer bacteriophage lysate on a 200-mesh copper carbon type-B electron microscope grid for one-two minutes. The lysate was wicked away and the grids were stained for 2 min using 15 μL of 2% phosphotungstic acid (pH = 7). Residual liquid was wicked away using Kimtech wipes and the grid was allowed to dry before being imaged. Bacteriophage structures in electron micrographs were measured using ImageJ (Abramoff et al., 2004). The average and standard deviation for each measurement was calculated from a minimum of four separate measurements.

### Burst Size

Burst size was calculated by performing single-infection assay as described by Delbruck (1945). The bacteria-bacteriophage mixture was allowed to adsorb for 10 min at a multiplicity of infection (MOI) of 100. The lysate was then removed at different time-intervals ranging from 1 to 6 h and diluted to avoid secondary infection. Soft agar plaque method was used to determine titers and a graph of 10 separate readings was plotted with their average titers and time.

### Host Range

Host range of all eight bacteriophages was determined using the soft agar plaque method (Hockett and Baltrus, 2017). For this, 50 μL of bacteriophage lysate dilutions were incubated with 500 μL of bacteria grown overnight for 30 min before plating in top agar. The plates were incubated with the top agar facing up at 25°C overnight for this assay. Seventeen bacterial strains including *E. amylovora* ATCC 29780 (Ritchie and Klos, 1976) as control were used including five other *E. amylovora* strains [Ea110 (Ritchie and Klos, 1976), GH9 (McGhee and Sundin, 2012), EaBH (McGhee and Sundin, 2012), RB02 (McGhee and Sundin, 2012), Ea273 (Sebaihia et al., 2010)], *Pantoea agglomerans* E325 (Pusey, 1997), *Pantoea vagans* C-91 (Ishimaru et al., 1987; Smits et al., 2010), *E. coli* K-12 BW 25113 (Datsenko and Wanner, 2000), *Salmonella enterica* LT2 (generously donated by John Roth lab), *Yersinia pestis* KIM6 (Fetherston et al., 1992; Gong et al., 2001)*, Enterobacter cloacae* ATCC 13047 (Ren et al., 2010), *Klebsiella pneumoniae* ATCC 10031 (Otman et al., 2007), *Bacillus subtilis* ATCC 6033 (Nakamura et al., 1999), *Cronobacter sakazakii* ATCC 29544 (Iversen et al., 2007; Gicova et al., 2014), standard clinical isolate *Pseudomonas aeruginosa* PA100 (Cartagena et al., 2007) and *Pseudomonas chlororaphis* ATCC 13985 (Conway et al., 1956). An average of two readings was taken to obtain bacteriophage titers post-infection.

### Computational Analysis and Genomic Comparison

Bacteriophages with any similarities to *Agrican357virus* genus were identified using a blastx analysis of their putative major capsid and terminase proteins, and the corresponding bacteriophage for all retrieved hits with a cutoff *e*-value of less than 1.00E-04 and 33% similarity were downloaded from GenBank. In addition, any bacteriophages that showed up in at least three qblast hits while annotating were also retrieved. These sequences were then used in Gepard (Krumsiek et al., 2007) to generate the dot plots of nucleic acid and protein sequences. PhamDB, a web interface (Lamine et al., 2016) was used for creating databases and Phamerator, (Cresawn et al., 2011) an open-source program was used to generate pham map of bacteriophage genomes. PhamDB uses kClust (Hauser et al., 2013) to cluster large protein sequence databases. The default settings of PhamDB were used in this comparison. Splitstree (Huson and Bryant, 2006) protein analysis was produced from the exported pham table of conserved proteins converted to a Nexus file using Janus^1^. The Average Nucleotide Identity (ANI) percentages comparing each of the *E. amylovora* bacteriophage genomes were calculated using MAFFT (Katoh et al., 2002) plugin in Geneious R8.1 (Kearse et al., 2012).

The evolutionary history was inferred by using the Maximum Likelihood method and Poisson correction model (Zuckerkandl and Pauling, 1965). The bootstrap consensus tree inferred from 100 replicates (Felsenstein, 1985) is taken to represent the evolutionary history of the taxa analyzed (Felsenstein, 1985). Branches corresponding to partitions reproduced in less than 50% bootstrap replicates are collapsed. The percentage of replicate trees in which the associated taxa clustered together in the bootstrap test (100 replicates) are shown next to the branches (Felsenstein, 1985). Initial tree(s) for the heuristic search were obtained automatically by applying Neighbor-Join and BioNJ algorithms to a matrix of pairwise distances estimated using a JTT model, and then selecting the topology with superior log likelihood value. This analysis involved 59 amino acid sequences. There were a total of 1302 positions in the final dataset. Evolutionary analyses were conducted in MEGA X (Kumar et al., 2018).

### Mass Spectrometry

Sample preparation was performed (Guttman et al., 2009) by diluting crude lysates of RAY and Deimos-Minion in TNE (50 mM Tris pH 8.0, 100 mM NaCl, 1 mM EDTA) buffer and adding RapiGest SF reagent (Waters Corp.) to a final concentration of 0.1%. Samples were then boiled for 5 min followed by addition of 1 mM (final concentration) of TCEP [Tris (2-carboxyethyl) phosphine] and incubated at 37°C for 30 min. Afterward, carboxymethylation of samples was done with 0.5 mg/ml of iodoacetamide for 30 min at 37°C followed by neutralization with 2 mM TCEP (final concentration). Trypsin (trypsin: protein ratio – 1:50) was used overnight at 37°C to digest the crude lysates prepared as above. The samples were treated with 250 mM HCl at 37°C for 1 h followed by centrifugation at 14000 rpm for 30 min at 4°C to degrade and remove RapiGest. The soluble fraction was then added to a new tube and Aspire RP30 desalting columns (Thermo Fisher Scientific) were used for extraction and desalting of the peptides.

High pressure liquid chromatography (HPLC) coupled with tandem mass spectroscopy (LC-MS/MS) using nano spray ionization was used to analyze Trypsin-digested peptides (McCormack et al., 1997). A TripleT of 5600 hybrid mass spectrometer (ABSCIEX) interfaced with nano-scale reversed-phase HPLC (Tempo) using a 10 cm-100-micron ID glass capillary packed with 5-μm C18 Zorbax^*TM*^ beads (Agilent Technologies, Santa Clara, CA) was used to perform the nano-spray ionization experiments. By using a linear gradient (5–60%) of ACN (Acetonitrile) at a flow rate of 250 μl/min for 1 h, peptides were eluted from the C18 column into the mass spectrometer The ACN gradient was created using these buffers: buffer A (98% H_2_O, 2% ACN, 0.2% formic acid, and 0.005% TFA) and buffer B (100% ACN, 0.2% formic acid, and 0.005% TFA). In a data-dependent manner MS/MS data were acquired in which the MS1 data was acquired for 250 ms at m/z of 400 to 1250 Da and the MS/MS data was acquired from m/z of 50 to 2,000 Da. For Independent data acquisition (IDA) parameters MS1-TOF 250 ms, followed by 50 MS2 events of 25 ms each. The IDA criteria; over 200 counts threshold, charge state of plus 2–4 with 4 s exclusion window. Finally, MASCOT^®^ (Matrix Sciences) was used to analyze the collected data and Protein Pilot 4.0 (ABSCIEX) was used for peptide identifications.

### Extracellular Polymeric Substance (EPS) Depolymerase Mediated Biofilm Degradation Assay

Soft agar plaque method (Hockett and Baltrus, 2017), as described previously in host range method, was used to detect the presence of halo zone on *P. vagans* strain C9-1 and *E. amylovora* ATCC 29780. The putative EPS-depolymerase from bacteriophage RAY was PCR amplified from lysate using primers designed to amplify the full length gp76. It was cloned by digesting with enzymes NdeI/SalI into a similarly digested pET15b. The resulting plasmid (JG1700) was amplified by transforming into *E. coli* DH5α and plated on LB-amp. Resulting colonies were PCR checked and were used to start overnight cultures and DH5α without plasmid pJG1700 was grown as a control. The protein was induced using IPTG and extracted by lysing cells via sonication. Post-sonication, cell debris was removed from both cultures by centrifuging at 12000 rpm and 4°C for 2 × 20 min. 10 μl of resulting supernatant was spotted on bacterial lawns of *P. vagans* strain C9-1 and *E. amylovora* ATCC 29780 embedded in top agar after plating for 2 h.

### Motif Identification and Analysis

MEME (Bailey et al., 2009) and FIMO (Grant et al., 2011) tools at public phage galaxy^2^ were used to scan bacteriophage genome of *Agrican357virus* for statistically significant motifs. Motifs found by MEME (Bailey et al., 2009) with *e*-value less than 1.00E-02 were selected by FIMO (Grant et al., 2011) to be searched for their coordinates and iterations in their respective genomes. User defined cut-off values (*P*-value <1.00E-03, *Q*-value <0.05), as described in Berg et al. (2016) were used to maximize the results. The location of the motifs within bacteriophage genomes was determined from the annotated GenBank files (Esplin et al., 2017).

## Results and Discussion

### Isolation and Characterization of Eight Closely Related Large Bacteriophages Infecting *E. amylovora*

Eight novel bacteriophages (Deimos-Minion, Special G, RAY, Simmy50, Bosolaphorus, Desertfox, Mortimer, and MadMel) that infect *E. amylovora* were plaque isolated and their genomes were subsequently sequenced and annotated as previously described (Esplin et al., 2017; Sharma et al., 2018). All eight bacteriophages have relatively large genomes with genome sizes of 271 to 275 kb (Table 1), which are comparable to the related bacteriophage Ea35-70 (271084 bp). These bacteriophages have correspondingly large putative proteomes, with 317 to 324 predicted ORFs. A search for tRNA’s using tRNA ScanSE (Lowe and Chan, 2016) suggests that RAY, Simmy50, Bosolaphorus, and Mortimer have 1 tRNA each coding for Asparagine, whereas no tRNA’s were detected for Deimos-Minion, Special G, MadMel, and Desertfox. No lysogeny related genes were identified (including integrase, excisionase or repressors). Their clear plaque morphology and ease in obtaining higher titers (∼10^8^–10^10^ pfu/ml) suggest they may be lytic bacteriophages, however, rigorous testing for bacterial lysogeny has not been performed.

**TABLE 1:** General characteristics of related bacteriophages Deimos-Minion (DM), RAY, Special G, Desertfox, MadMel, Mortimer, Bosolaphorus, Simmy50, and Ea35-70 that infect *E. amylovora* ATCC 29780.

**Phage name**	**GenBank accession**	**Genome length (bp)**	**Sample type**	**Conserved domains**	**ORFs (tRNAs)**	**Gene differences compared to DM**	
						**Extra genes**	**Missing**
						
Deimos-Minion (DM)	KU886225	273,501	fruit	39	324		
RAY	KU886224	271,182	leaves, stem	39	317 (1)	0	gp49, gp50, gp90, gp91, gp166, gp234
Special G	KU886222	273,224	branches, blossoms	41	321	gp63, gp203, gp231	gp90, gp91, gp111, gp166, gp234
Desertfox	MG655268	272,458	soil	39	320	gp106, gp231, gp256,	gp48, gp50, gp90, gp91, gp111, gp234
MadMel	MG655269	275,000	soil	41	321	gp62, gp202, gp230	gp90, gp91, gp111, gp252
Mortimer	MG655270	273,914	−	40	324 (1)	gp62, gp110, gp238, gp261	gp48, gp117, gp234
Bosolaphorus	MG655267	272,228	orchard dirt	39	320 (1)	gp223	gp48, gp90, gp91, gp234
Simmy50	KU886223	271,088	bark	39	322 (1)	gp8, gp63, gp209, gp210	gp51, gp90, gp91, gp166, gp234
Ea35-70	KF806589	271,084	soil	36	318 (1)	gp61, gp115, gp224	gp86, gp93, gp120, gp166, gp232, gp234, gp252

*Sample type is as reported by collectors, no sample type was recorded for Mortimer. Due to the high conservation of this family, differences in encoded genes is also provided with missing genes numbered with respect to Deimos-Minion.*

### Electron Microscopy Reveals Myovirus Structure for Five *E. amylovora* Bacteriophages

**FIGURE 1:**
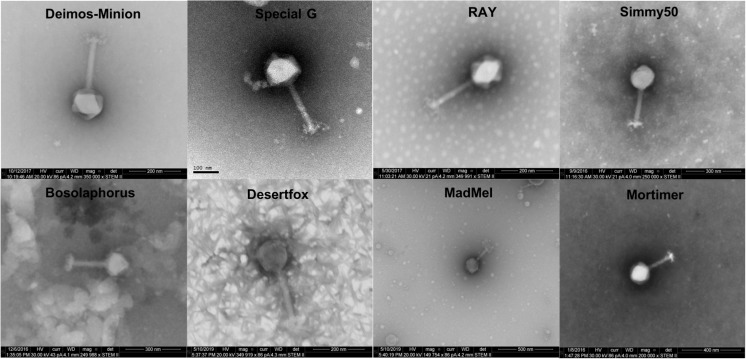
Electron microscopy STEM images of Deimos-Minion, Special G, RAY, Simmy50, Bosolaphorus, Desertfox, MadMel, and Mortimer revealed *Myoviruses* with long contractile tails.

Deimos-Minion, Special G, RAY, Simmy50, Mortimer, MadMel, Desertfox, and Bosolaphorus were all found to be similarly sized *Myoviridae*, having contractile tails (average size 159 + −11.4 nm), a tail sheath (average size 78.5 + −9.28 nm), visible tail fibers, and large capsids (average size 128 + −5.96 nm) (Figure 1). This morphology is supported by their protein-based relationships to other jumbo *Myoviridae* discussed below. Due to apparent similarity within these bacteriophages, only RAY’s morphological calculations are listed but all eight of these bacteriophages were imaged extensively.

### Host Range and Burst Size

Bacteriophages of the *Agrican357virus* family were tested for activity against seventeen different bacterial strains. Out of these, fifteen were from the *Enterobacteriales*- *P. agglomerans* E325 (Pusey, 1997), *P. vagans* C-91 (Ishimaru et al., 1987; Smits et al., 2010), *E. coli* K-12 BW 25113 (Datsenko and Wanner, 2000), *S. enterica* (generous donation by roth lab), *Y. pestis* KIM6 (Fetherston et al., 1992; Gong et al., 2001), *E. cloacae* ATCC 13047 (Ren et al., 2010), *K. pneumoniae* ATCC 10031 (Otman et al., 2007), *B. subtilis* ATCC 6033 (Nakamura et al., 1999), *C. sakazakii* ATCC 29544 (Iversen et al., 2007; Gicova et al., 2014), *E. amylovora* Ea110 (Ritchie and Klos, 1976), *E. amylovora* GH9 (McGhee and Sundin, 2012), *E. amylovora* EaBH (McGhee and Sundin, 2012), *E. amylovora* RB02 (McGhee and Sundin, 2012), *E. amylovora* Ea273 (Sebaihia et al., 2010), *E. amylovora* ATCC 29780 (control) (Ritchie and Klos, 1976), and two from *Pseudomonadaceae*- *P. aeruginosa* PA100 (Cartagena et al., 2007) and *P. chlororaphis* ATCC 13985 (Conway et al., 1956). *Enterobacteriales* strains were chosen due to being members of the same bacterial order as *Erwinia*, whereas *Pseudomonadaceae* strains were the hosts of bacteriophages related to the *Agrican357virus* bacteriophages based on protein BLAST. Our current analyses displayed that *Agrican357virus* bacteriophages infect all *Erwinia* strains (with the exception of Special G and Mortimer that failed to infect GH9 and EaBH, respectively) as well as closely related genera also commonly found on plants– *P. agglomeran*s (Deletoile et al., 2009) and *P. vagans* (Palmer et al., 2016; Table 2).

**TABLE 2:** Host range analysis of eight *Agrican357virus* bacteriophages.

**Bacterial strains (strain number)**	**Bacteriophages**							
	
	**Deimos-Minion**	**RAY**	**Special G**	**Desertfox**	**MadMel**	**Mortimer**	**Bosolaphorus**	**Simmy50**
*E. amylovora (ATCC 29780)*	5.20E+09	7.80E+09	3.40E+09	2.56E+07	5.42E+08	2.87E+06	3.29E+04	4.33E+08
*E. amylovora GH9*	3.03E+10	3.90E+09	−	1.77E+07	5.00E+06	3.49E+05	5.09E+04	4.15E+08
*E. amylovora EA110*	6.60E+09	5.20E+09	4.52E+09	9.00E+06	3.63E+08	5.26E+06	5.65E+04	8.97E+08
*E. amylovora EaBH*	5.70E+09	4.40E+09	2.60E+09	1.06E+07	5.78E+08	−	6.04E+04	3.00E+08
*E. amylovora RB02*	3.25E+09	4.50E+09	1.84E+08	3.65E+07	4.47E+08	1.06E+06	5.03E+04	2.42E+08
*E. amylovora 273*	1.04E+10	9.75E+09	1.45E+07	2.36E+07	4.37E+08	5.39E+06	6.15E+03	1.62E+08
*E. amylovora (ATCC 29780)*	6.10E+09	6.90E+09	3.94E+08	3.20E+07	6.04E+08	2.05E+06	5.60E+04	2.61E+08
*P. vagans (C9-1)*	3.14E+10	2.64E+10	1.00E+11	5.01E+07	2.05E+09	6.39E+06	4.05E+03	4.95E+09
*P. agglomerans (E325)*	3.10E+10	9.30E+09	2.60E+10	5.80E+06	2.67E+09	2.90E+06	2.79E+04	4.48E+09
*P. chlororaphis (ATCC 13985)*	–	−	−	–	−	−	–	−
*E. coli k-12 (BW 25113)*	–	−	−	–	−	−	–	−
*B. subtilis (ATCC 6033)*	–	−	−	–	−	−	–	−
*C. sakazakii (ATCC 29544)*	–	−	−	–	−	−	–	−
*K. pneumoniae (ATCC 10031)*	–	−	−	–	−	−	–	−
*S. enterica (Roth lab)*	–	−	−	–	−	−	–	−
*E. cloacae (ATCC13047)*	–	−	−	–	−	−	–	−
*P. aeruginosa (PA100)*	–	−	−	–	−	−	–	−
*Y. pestis (KIM6)*	–	−	−	–	−	−	–	−

*Host range tests on *Agrican357virus* displays infection of *E. amylovora* strains ATCC 29780 (control), *GH9, Ea110, EaBH, RBO2, Ea273, P. agglomerans* (E325), and *P. vagans* (C9-1) only. Bacteriophages Special G and Mortimer failed to infect strain *EaGH9* and *EaBH*, respectively. All other bacterial strains remained uninfected. Symbol “–”, no infection based on plaque assay under laboratory conditions, dark and light gray indicates bacterial strains and bacteriophages, respectively. Plaque forming units (pfu) should be compared to the ATCC strain, because the same amount of the same lysate was used to infect each strain.*

**FIGURE 2:**
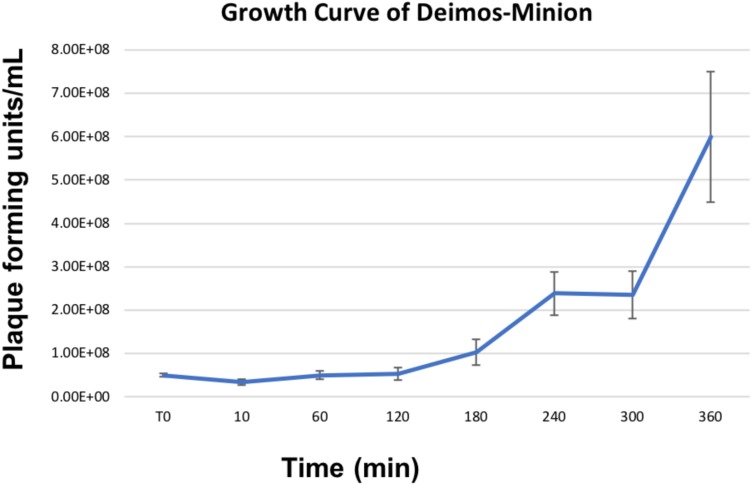
Growth Curve of Deimos-Minion with host *E. amylovora* ATCC 29780 by plaque assays shows first burst at ∼4 h and second burst at ∼6 h.

Owing to the large nature of *Agrican357virus* bacteriophages, we investigated the burst size of bacteriophage Deimos-Minion on *E. amylovora* strain ATCC 29780. Burst size studies suggested that when infected at MOI of 100 Deimos-Minion has burst size of 4.6–4.9 with latent period of 3–4 h before the first burst (Figure 2) under the laboratory growth conditions used herein, consistent with their large size. As seen in Figure 2, a second burst is appearing at the end of this 6 h period. The observed burst size (∼5) was confirmed with bacteriophage RAY (data not shown) and is consistent with other large *Myoviridae* in that *P. aeruginosa* bacteriophage KTN4 has a reported burst of 6–8 and may be due to the need to build internal cellular structures for the Jumbo viruses to be built (Danis-Wlodarczyk et al., 2016), or due to sub-optimal assay conditions for proliferation.

### Genomic and Evolutionary Characteristics

**FIGURE 3:**
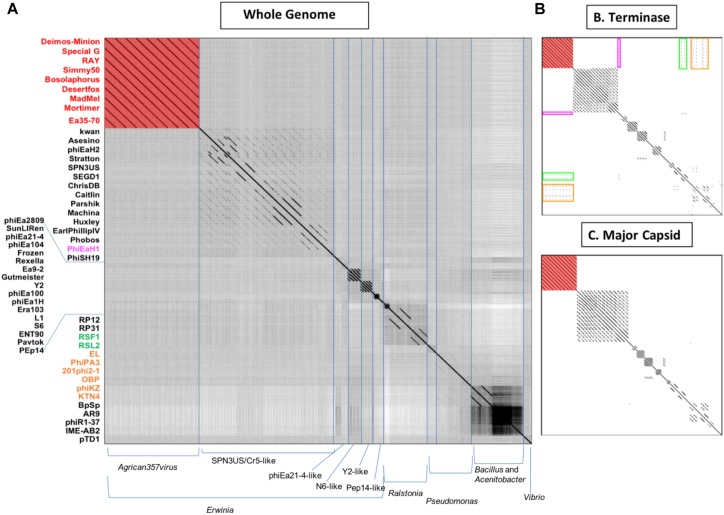
Whole-genome nucleotide **(A)** and protein terminase **(B)** or major capsid protein **(C)** dot plot analysis reveals a fairly isolated cluster of bacteriophages that includes Deimos-Minion, Special G, RAY, Simmy50, Bosolaphorus, Desertfox, MadMel, Mortimer and Ea35-70. Dot plots were constructed using Gepard.

To determine the overall genomic and proteomic similarity of our eight novel bacteriophages to available bacteriophages in GenBank, related bacteriophages were identified by BLAST (qblast) using each of the putative gene products encoded by RAY. The bacteriophages with *e*-values below 1.00E-04 and above 33% identity that were identified in three or more BLAST searches were then compared using Gepard dot plot (Krumsiek et al., 2007), average nucleotide identity (ANI analysis) (Lassmann and Sonnhammer, 2005), and BLAST alignment (Altschul et al., 1990). Dot plots were constructed using whole genome sequences, major capsid protein amino acid, and terminase amino acid sequences (Figures 3A–C, respectively). While looking at the results of the whole genome dot plot, all eight of our bacteriophages show no similarity to any other bacteriophages used in the dot plot except for very close similarity to Ea35-70 (KF806589) (Yagubi et al., 2014), an *Erwinia* bacteriophage isolated in Canada in 2014 (see Figure 3A). In addition, their average nucleotide identity (ANI) using Geneious (Kearse et al., 2012) was remarkably high >94% (see Supplementary Table S1). These results indicate that these eight bacteriophages Deimos-Minion, Simmy50, RAY, Special G, Bosolaphorus, Desertfox, MadMel, and Mortimer along with Ea35-70 make a distinct family of bacteriophages, consistent with the International committee on taxonomy of viruses’ classification as new species of a new genus *Agrican357virus* in the family *Myoviridae* of order *Caudovirales* (Adams et al., 2017).

The major capsid protein (MCP) and terminase proteins are two of the most conserved proteins in bacteriophage genomes and have been used to group bacteriophages in families by single gene analysis (Smith et al., 2013). In order to identify distant bacteriophage relatives, a proteomic comparison of these bacteriophages was performed using terminases (see Figure 3B) and MCPs (see Figure 3C) by Gepard dot plot (Krumsiek et al., 2007). The same bacteriophage order from the whole genome dot plot (Figure 3A) was used in these dot plots. Whole genome and terminase dot plots both displayed limited synteny between *Agrican357virus* bacteriophages and *Erwinia* bacteriophage phiEaH1 (4.00E-155 from blastp of terminase) indicating this bacteriophage as the closest known relative from *Erwiniaceae*. In contrast, little similarity to *Pseudomonas* bacteriophages phiKZ (8.00E-156), KTN4 (8.00E-156), phiPA3 (2.00E-149), 201phi2-1 (5.00E-140), OBP (2.00E-101), EL (3.00E-77), and *Ralstonia* bacteriophages RSF1 (9.00E-122) and RSL2 (3.00E-120) can be seen in the terminase dot plot which was not apparent in the whole genome and major capsid protein dot plots. All of these bacteriophages are distantly related jumbo *Myoviridae*.

**FIGURE 4:**
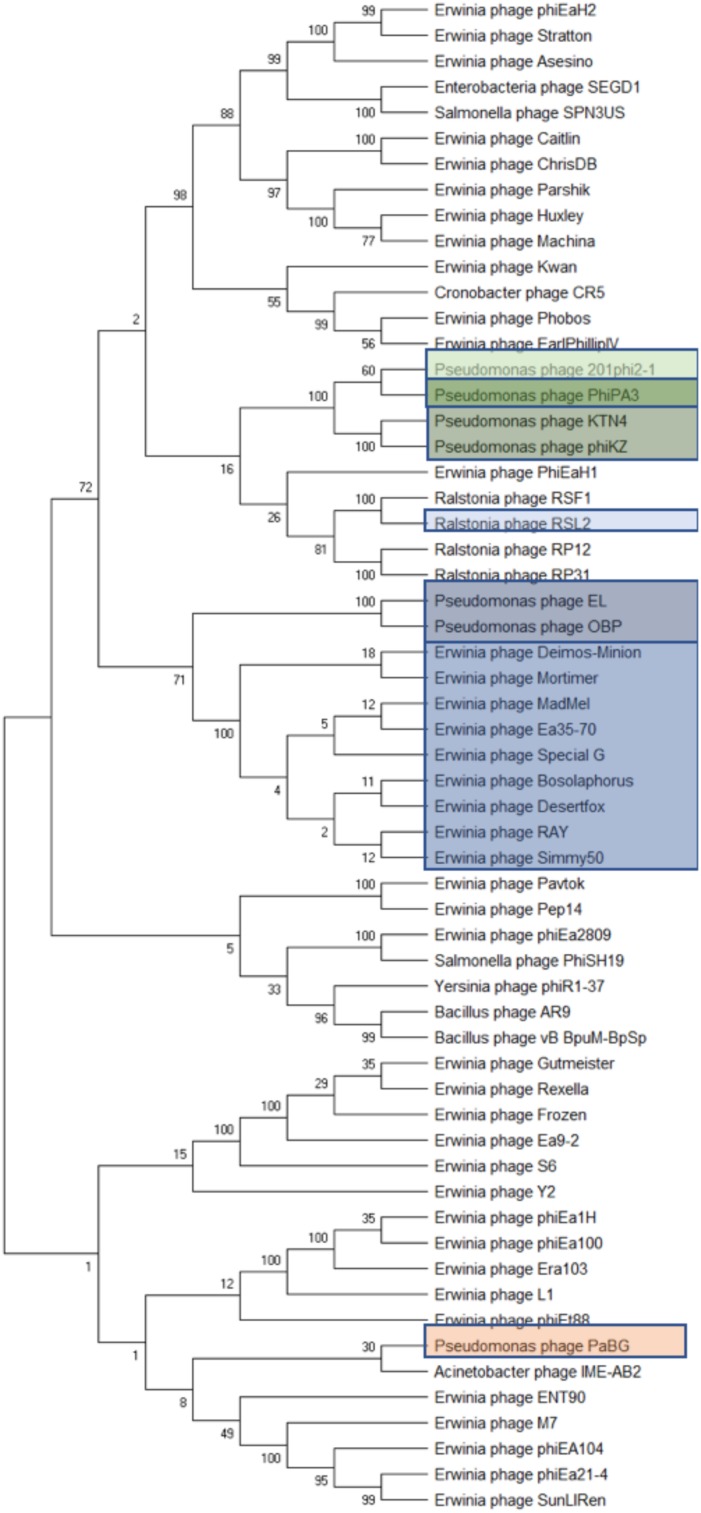
Phylogenetic analyses of phage terminase proteins supports the relationships depicted by dot plot analysis of the *Agrican357virus* bacteriophages. The evolutionary history was inferred using the Maximum Likelihood method and Poisson correction model in MEGA X. Branches corresponding to partitions reproduced in less than 50% bootstrap replicates are collapsed where 100 was set to be initial bootstrapping value.

The two subunits of terminase protein; large and small, are an essential part of DNA packaging (Kuebler and Rao, 1998; Mesyanzhinov et al., 2002). All eight of our *Agrican357virus* bacteriophages have a putative terminase gene with identical amino acid sequences: Deimos-Minion gp189, Special G gp185, RAY gp183, Simmy50 gp186, Desertfox gp184, Bosolaphorus gp185, MadMel gp185, and Mortimer gp188. This protein is also present in Ea35-70 gp181. This indicates that it is a highly conserved protein for this family. Considering the similarity between these bacteriophages, it can be inferred that all nine bacteriophages of *Agrican357virus* may have headful packaging (Figure 4). In support of this conclusion, blastp results demonstrated a match with *Pseudomonas* bacteriophage phiKZ with an *e*-value of 8.00E-156, a terminase large subunit from *Erwinia* bacteriophage PhiEaH2 with an *e*-value of 6.00E-122 and a terminase large subunit of *Pseudomonas* bacteriophage 201phi2-1 with an *e*-value of 5.00E-140. Bacteriophages phiKZ, phiEaH2 and 201phi2-1 are all known to have headful packaging (Merrill et al., 2016). In addition to blastp, bacteriophage termini and packaging mode for six bacteriophages (excluding Deimos-Minion and Special G) was also determined using randomly fragmented next-generation sequencing (NGS) data with the help of software PhageTerm (Garneau et al., 2017)^3^. PhageTerm analysis indicated that RAY, MadMel, Desertfox, Bosolaphorus, Simmy50, and Mortimer have headful packaging without a pac site. Thus, the headful packaging strategy is supported by terminase homology and NGS sequencing data.

### Proteomic Analysis of the *Agrican357virus* Family

Due to great similarity between these bacteriophages we randomly chose RAY as a representative for the protein classification. Proteomic analysis of RAY reveals the novel nature of these bacteriophages in that of 318 proteins, 202 proteins were considered to be novel with no BLAST hit (the *e*-value cutoff was <1.00E-04), 50 were hypothetical proteins with BLAST hits, and 67 were proteins with a putative function based on their BLAST hit (Supplementary Figure S1A). Thus, over half of the proteins had no BLAST hit outside of the *Agrican357virus* bacteriophages. These proteins represent a considerable proteomic “dark matter” (Hatfull, 2015), and underscore the vast biological richness harbored in bacteriophages. Of the 67 proteins with predicted function, a majority appear to be structural proteins (∼41%), and DNA metabolism proteins (approximately 41%) (Supplementary Figure S1B).

**FIGURE 5:**
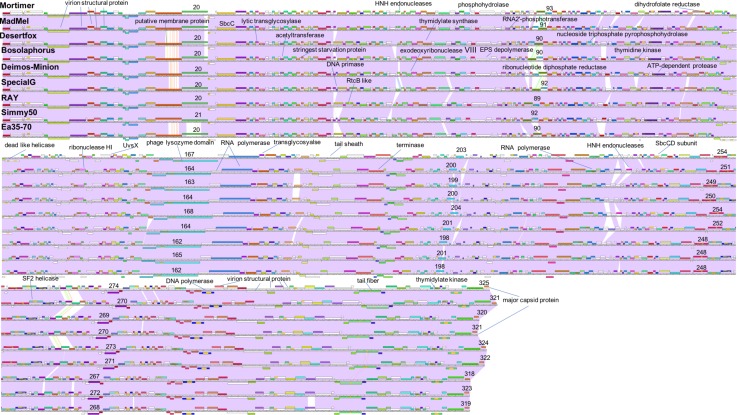
Whole genome Phamerator map of *E. amylovora* bacteriophages illustrates the high similarity of bacteriophages Mortimer, MadMel, Desertfox, Bosolaphorus, Deimos-Minion, Special G, RAY, Simmy50, and Ea35-70. Bacteriophages were mapped using Phamerator and arranged based on highest protein similarity. Violet shading between genomes indicates genome nucleotide homology (with standard *e*-value cutoff of 1.00E-04) and the ruler indicates genome base pairs, while white spaces indicate areas without significant nucleotide similarity. Boxes above and below the genome ruler indicate ORFs going in the forward and reverse direction, respectively. They are labeled with predicted function, occasionally numbered, and colored to indicate protein homologs between the bacteriophages.

The computer program Phamerator (Cresawn et al., 2011) was used to compare the entire genomes of the nine *Agrican357virus* bacteriophages that infect *E. amylovora*: Deimos-Minion, Special G, RAY, Simmy50, Bosolaphorus, Desertfox, MadMel, Mortimer, and Ea35-70 (Figure 5). Despite their large size, these genomes display remarkable nucleotide sequence and proteomic conservation (>94% ANI, see Supplementary Table S1). The genomes encode recognizable structural and enzymatic bacteriophage proteins vital to the bacteriophage life cycle, including terminase proteins, major capsid proteins, and tail fiber proteins as well as proteins involved in DNA transcription and translation, such as helicase proteins, DNA polymerase, and RNA polymerase. Though the genomes of these nine bacteriophages are virtually identical, a few genes are differentially present across these bacteriophage genomes. Most of these are hypothetical proteins, however, HNH endonucleases also differed consistently between the *Agrican357virus* bacteriophages. HNH endonucleases are proteins that splice DNA and assist in the movement of introns and other intron-like sequences (Chevalier and Stoddard, 2001). Deimos-Minion has two such HNH endonucleases, gp93 and gp234 that do not appear to be homologs based on protein similarity. Protein BLAST results of gp93 show that the HNH endonuclease is also found in bacteriophages Bosolaphorus, Desertfox, MadMel, RAY, Simmy50, Special G and Ea35-70, and is similar to those found in some *Pseudomonas* bacteriophages (phiKZ and KTN4) as well as both Gram-negative and Gram-positive strains of bacteria. However, only the HNH endonuclease domain (∼amino acid 58-109 of bp93) is primarily conserved, the remaining 278 amino acid protein is not conserved in bacteria. On the other hand, homologs of HNH endonuclease gp234 are only found in Deimos-Minion and MadMel, as well as several Gram-positive and Gram-negative bacteria. Genomes of Deimos-Minion, Desertfox and MadMel also displayed a reversed order of two proteins (gp93-gp94 in Deimos-Minion, gp88-gp89 in Desertfox and gp90-gp91 in MadMel) when compared to similar proteins in other bacteriophages of this family. The proteins involved are HNH endonuclease and ribonucleotide reductase. To search for repetitive sequences in the genome which may be involved in recombination, MEME (Bailey et al., 2009) and FIMO (Grant et al., 2011) were used to locate motifs in the genomes of all eight of our *Agrican357virus* bacteriophages. Several common and unique motifs were discovered, however, they had poor *e*-values with little or no significance and were not followed further.

**FIGURE 6:**
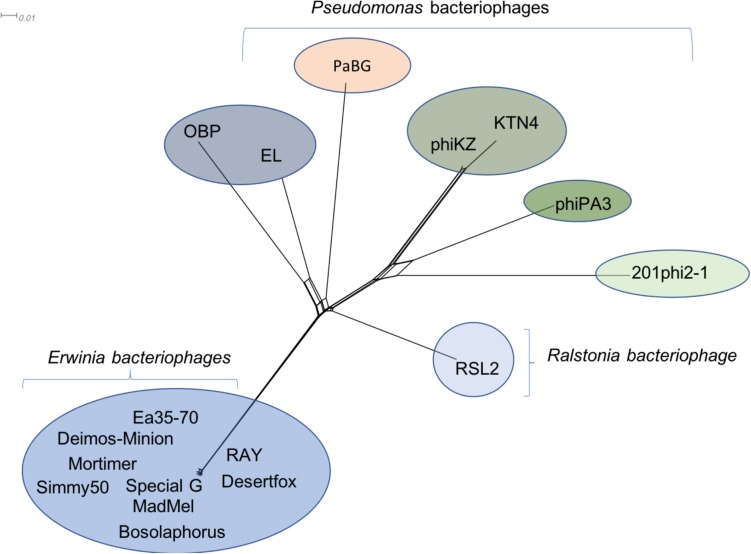
Protein-conservation analysis displayed by Splitstree of the *Agrican357virus* genus with related jumbo *Myoviridae* bacteriophages reveals *Agrican357virus* as a distant evolutionary group.

Due to the large size of these bacteriophages, and their terminase similarity to bacteriophage phiKZ, these bacteriophages likely belong to the jumbo bacteriophages (Hertveldt et al., 2005; Yuan and Gao, 2017) making it no surprise that the structural proteins are found in other bacteriophages. Along with hypothetical proteins, the proteins that are conserved with other phiKZ-like jumbo bacteriophages include: RNA polymerase beta subunit, nuclease RtcB-like, SbcC like, helicase, virion structural proteins, tail fiber, tail sheath, lysozyme domain, terminase, and major capsid protein. A splitstree analysis showing the relationship of the related jumbo bacteriophages by protein conservation is displayed in Figure 6. This protein-based tree suggests seven groups of related jumbo *Myoviridae* bacteriophages, with the *Agrican357virus* group as the most distant group. It further confirms that proteins of *Agrican357virus* family are more similar to proteins from *Pseudomonas* bacteriophages EL and OBP and *Ralstonia* bacteriophage RSL2 than to other *Enterobacteriales* bacteriophages.

### Mass Spectrometry Validates 27 Hypothetical Proteins as Proteins of Unknown Function

Further analysis of Deimos-Minion and RAY genomes via mass spectrometry (MS) detected several novel proteins, promoting the status of 27 proteins from hypothetical proteins to proteins of unknown function. In RAY and Deimos-Minion genomes collectively, MS analysis identified seventeen proteins with a putative function, eighteen novel hypothetical proteins specific to this bacteriophage family and nine hypothetical proteins (seven known bacteriophage proteins and two other) with blastp hits to other bacteriophages (Table 3). The majority of proteins found through MS are novel hypothetical proteins found only in this family, followed by putative bacteriophage structural proteins, hypothetical bacteriophage proteins, proteins with putative functions and other hypothetical proteins (see Table 3). This analysis agrees with our predicted conservation of proteins depicted through Phamerator analyses.

**TABLE 3:** Mass Spectrometry reveals 27 Hypothetical Proteins as Proteins of Unknown Function.

**RAY**	**DM**	**Putative function**	**Retrieval #**		**% coverage**	
			**RAY**	**DM**	**RAY**	**DM**
**Putative bacteriophage structural proteins**						
	gp323	putative major capsid protein		4		62.65
gp178		putative virion structural protein	35		40.2	
gp154		putative virion structural protein	57		28.6	
gp179	gp185	putative tail sheath protein	105	45	22.	43.45
gp18		putative virion structural protein	61		18.	
	gp308	putative tail fiber protein		106		51.21
	gp9	putative virion structural protein		146		23.16
	gp19	putative virion structural protein		153		10.92
	gp188	putative virion structural protein		121		10.14
gp293		putative virion structural protein	104		31.89	
**Putative enzymatic proteins**						
gp76	gp79	putative EPS-depolymerase	58	89	23.6	20.16
gp162		putative phage-related lysozyme	94		29.2	
gp102	gp107	putative nucleotide triphosphatase	103	72	25.6	39.53
	gp127	putative dihydrofolate reductase		171		12.1
	gp23	putative SbcC-like protein		169		25.18
	gp228	putative DNA-directed RNA pol.		67		41.82
	gp94	putative ribonucleotide diphosphate reductase beta subunit		91		10.32
**Novel hypothetical proteins found only in this bacteriophage family**						
gp281	gp287	novel hypothetical protein	6	61	33.5	36.31
gp295	gp301	novel hypothetical protein	9	64	23.8	29.11
gp287		novel hypothetical protein	17		71.7	
gp185	gp191	novel hypothetical protein	18	68	66.0	68.49
gp188		novel hypothetical protein	33		35.5	
gp186		novel hypothetical protein	41		16.8	
gp196	gp202	novel hypothetical protein	44	137	34.9	21.7
gp55		novel hypothetical protein	46		37.3	
gp316		novel hypothetical protein	47		42.6	
gp110	gp114	novel hypothetical protein	49	116	21.6	33.99
gp298	gp304	novel hypothetical protein	50	70	42.8	27.72
gp173	gp179	novel hypothetical protein	55	55	58.1	61.49
gp166		novel hypothetical protein	78		40.9	
gp75		novel hypothetical protein	92		29.0	
gp99		novel hypothetical protein	62		28.0	
gp207	gp212	novel hypothetical protein	95	95	4.7	17.13
gp98	gp103	novel hypothetical protein	97	133	6.8	9.74
	gp140	novel hypothetical protein		84		18.49
**Hypothetical bacteriophage proteins**						
gp222	gp227	hypothetical phage protein	23	87	33.4	39.1
gp240	gp246	hypothetical phage protein	34	81	32.3	57.42
gp301	gp307	hypothetical phage protein	54	93	32.26	58.8
gp202		hypothetical phage protein	73		43.3	
gp292		hypothetical phage protein	79		37.9	
	gp251	hypothetical phage protein		88		25.88
	gp224	hypothetical phage protein		129		33.33
**Other hypothetical proteins**						
gp273		hypothetical protein	68		18.7	
gp41		hypothetical protein	56		34.2	

*Peptides detected by LC/MS/MS of a crude bacteriophage lysate of RAY and/or Deimos-Minion. Columns provide the gene product number corresponding to the peptide(s) detected, the putative function of the protein, the mass spectrometry retrieval number (which may reflect abundance), and the percent coverage for the protein. Gene products are grouped by putative function when available, and then by conservation. Deimos-Minion is abbreviated to DM.*

### Biofilm Degradation (EPS) Assays Suggest Specificity for *Pantoea*

**FIGURE 7:**
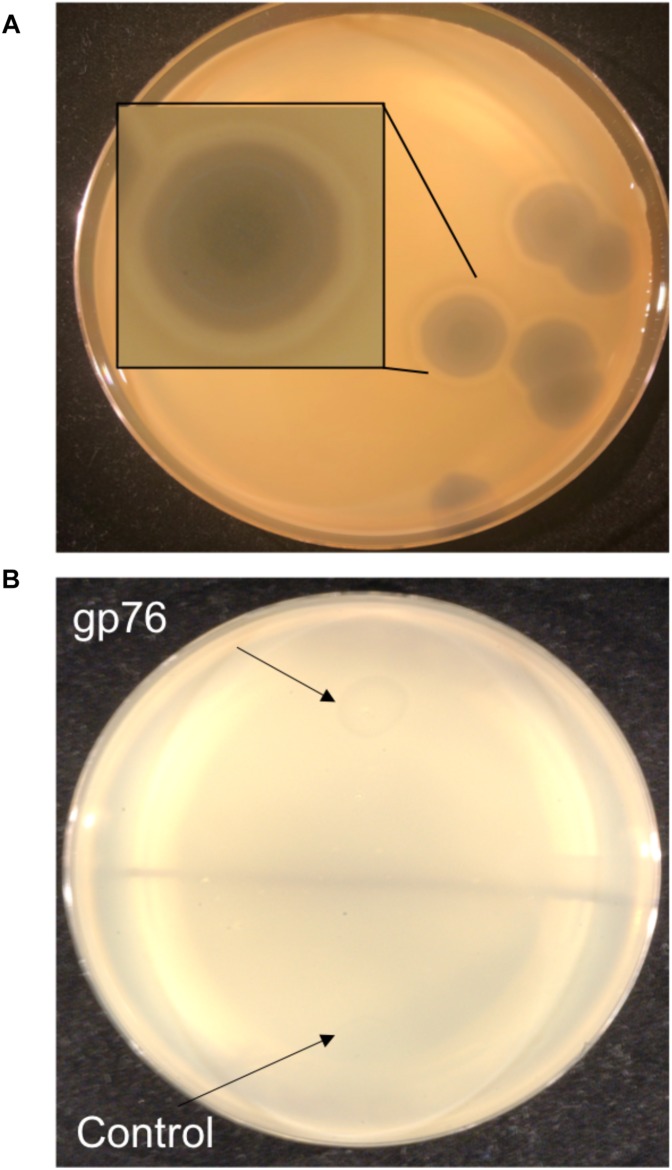
Halo formation on *P. vagans* by RAY **(A)** and bio-film degradation activity of gp76 on *P. vagans* **(B)**.

Enzymatic proteins like extracellular polysaccharide (EPS) depolymerase and phage-related lysozyme are few of the annotated proteins with putative functions which were also predicted via mass spectrometry. EPS depolymerase (Kim and Geider, 2000) is an enzyme that degrades EPS and phage-related lysozyme is shown to lyse the bacterial cell wall (Emrich and Streisinger, 1968). It has been shown that halo formation on the host could be a result of biofilm degradation activity (Cornelissen et al., 2012; Majkowska-Skrobek et al., 2016). The presence of halo zone after in infections of *Agrican357virus* family was first observed on *P. vagans* strain C9-1 (Figure 7A). To investigate further the EPS- depolymerase gene was cloned into a plasmid pJG1700, amplified using *E. coli* DH5α, and spotted on *P. vagans* stain C-91 and *E. amylovora* strain ATCC 29780 (Figure 7B). Lysate from a similarly grown and prepared DH5α culture was used as a control. The clearing is indicative of EPS depolymerase activity on *P. vagans*. This activity was not seen on *E. amylovora* ATCC 29780.

### Structural Prediction Supports the Putative Function of Several Proteins

To further understand *Agrican357virus* and verify their protein functions, we studied proteins involved in DNA metabolism (∼45%), the largest group of functional proteins conserved in the *Agrican357virus*. Multiple mechanisms for DNA regulation and repair are evident with the presence of proteins that are hypothesized to aid DNA synthesis, repair, and recombination. These proteins may increase the stability and survival of these jumbo bacteriophages (Supplementary Table S2 and Supplementary Figure S2). In order to proliferate in host cells, bacteriophages need to be equipped with proteins that allow them to reproduce effectively. Although many bacteriophages harbor proteins for DNA damage repair and DNA reproduction inside a host bacteria cell, these large bacteriophages may require extremely viable progeny due to lower burst sizes (∼4.6 functional virions compared to thousands reported for other bacteriophages).

Two proteins with a conserved domain found in the nine *Agrican357virus* bacteriophages are SbcC and a SbcCD nuclease (see Supplementary Figure S2A). The ability of SbcC and SbcCD to regulate and repair DNA has been shown to be essential for the stability and proliferation of some bacteriophages (Connelly et al., 1998). During DNA replication, palindromic sequences will create hairpin-like structures that can inhibit the progression of DNA polymerase (Leach et al., 1997). SbcC and SbcCD proteins work together to cleave both double- stranded and single-stranded DNA, and have been shown to recognize and specifically cleave hairpin structures. This breaks down the replication fork, allowing the genome to be repaired through recombination, so replication can proceed (Leach et al., 1997; Connelly et al., 1998). The proteins SbcC and SbcCD nucleases preserve the viability of the genome by allowing replication without excising the palindromic sequences (Leach et al., 1997). There are many types of DNA damage that may occur within a genome, making recombination and repair of DNA important, such as mutations due to UV damage. UV damage creates kinks or abnormalities within a genome, and prohibits proliferation. Exodeoxyribonuclease VIII breaks double stranded DNA, and degrades a genome on both 5′ ends (Joseph and Kolodner, 1983; Kolodner et al., 1994). This allows the kinked and abnormal portions of a genome to be straightened and repaired through homologous recombination. Additionally, exodeoxyribonuclease VIII does not require ATP to perform DNA repair, enabling repair of the genome even in low-energy environments where the bacteriophage does not have access to ATP (Kolodner et al., 1994). We hypothesize that exodeoxyribonuclease VIII enables the bacteriophages to remain stable despite mutations from UV damage. However, unique from our other predicted structure alignments, the protein from RAY does not match up well with other exodeoxyribonuclease VIII homologs (see Supplementary Figure S2B). It is possible that since these proteins do not have the same protein folding and alignment, they may not have the same function but a related, adapted function.

In the *Agrican357virus* bacteriophages, there are several encoded proteins with conserved domains of the thymidylate kinase and thymidine kinase (see Supplementary Table S2). Structural prediction and alignment confirm these proteins as likely thymidine kinases (see Supplementary Figures S2C,D), a necessary step due to the distant relationship (low *e*-values) of *Agrican357virus* bacteriophage proteins when compare to other biological entities. Thymidine kinase is an enzyme that catalyzes the phosphorylation of thymidine monophosphate (Kokoris and Black, 2002). Thymidylate kinase then catalyzes the phosphorylation of thymidine diphosphate (Doharey et al., 2016), which is an essential precursor for DNA (Chaudhary et al., 2013). Therefore, these proteins are regulatory enzymes that make bacteriophage cell growth and survival possible by aiding proliferation through the synthesis of DNA (Chaudhary et al., 2013; Cui et al., 2013; Doharey et al., 2016). Other proteins shown in Supplementary Figures S2E,F are putative UvsX recombinase and a putative SF2 helicase with conserved helicase domain known as UvsW, which finishes the recombination (Madej et al., 1995; Gajewski et al., 2011). UvsX and UvsW are proteins that have been known to work together to repair broken replication forks through homologous recombination (Kadyrov and Drake, 2004; Maher and Morrical, 2013). Homologous recombination is one of the most efficient ways to have error free DNA repair, and is beneficial to bacteriophages to have this repair mechanism. These repair mechanisms would be important to the bacteriophages because it would not only help repair broken replication forks but it would also help repair damaged or broken DNA (Kadyrov and Drake, 2004; Maher and Morrical, 2013). It has been shown that the absence of UvsX increases UV sensitivity (Kadyrov and Drake, 2004).

## Conclusion

*Agrican357virus* genus of bacteriophages are *Myoviridae* with dsDNA, large capsids, long contractile tails and high GC content. Their genomes are nearly identical (>94% ANI). All three dot plots (whole genome, major capsid protein, and terminase protein) show no close similarity between the *Agrican357virus* family and any of the other bacteriophages on NCBI (see Figures 3A–C). We have also found that the *Agrican357virus* cluster is more closely related to bacteriophages infecting *Pseudomonas* and *Ralstonia*, than those infecting *E. amylovora*. The contrast that we observe between this cluster of bacteriophages and the distantly related bacteriophage analyzed by dot plot contributes valuable information about evolutionary relationships between these other clusters (see Figure 3), suggesting the distant relationship may emphasize the importance of ecological niche, since most other *Enterobacteriales* bacteriophages isolated infect animal pathogens rather than plant pathogens. It may also, however, simply indicate the abundance of unstudied bacteriophages. The *Agrican357virus* family of bacteriophages is a novel family, with very low similarity to any other viruses, providing approximately 250 novel proteins to add to the viral dark matter that have no homolog by blastp (Hatfull, 2015). To understand a bacteriophage, it is vital to understand the encoded proteome. A bacteriophage’s proteins determine how effectively it can infect bacteria, and how stable and safe it would be to use in a phage cocktail (a mixture of bacteriophages used together for phage therapy). Of the proteins with predicted function, this family encodes primarily DNA metabolism and repair proteins. Since the bacteriophage host, *E. amylovora*, is found primarily on the blossoms of fruit trees of the *Rosaceae* family, these proteins may be particularly vital due to the onset of UV radiation including putative thymidine and thymidylate kinases which aid the production of the nucleotide thymine for DNA synthesis (Doharey et al., 2016), putative SbcC and SbcCD proteins which protect against DNA damage by cleaving harmful hairpin structures during replication (Connelly et al., 1998), putative exodeoxyribonuclease VIII which makes double stranded DNA breaks to help repair DNA damage at low energy (Joseph and Kolodner, 1983), and putative UvsX recombinase and putative SF2 helicase which aid in repair and recombination of DNA (Maher and Morrical, 2013). The small burst size we report herein for these jumbo bacteriophages (∼4.6 functional virions), may require a high level of fidelity to ensure success in the environment.

A paper published in 2003 on evolutionary pathways of *P. aeruginosa* bacteria demonstrated that phiKZ-like bacteriophages have a very broad host range (Krylov et al., 2003). In 1995, Campbell et al. (Campbell et al., 1995) isolated bacteriophages from barley rhizosphere that infected *Pseudomonas* spp. other than *P. aeruginosa*. These bacteriophages displayed great morphological similarity to phiKZ-like bacteriophages despite low genomic similarity (Mesyanzhinov et al., 2002; Krylov et al., 2003; Sokolova et al., 2014). Similarly, *Agrican357virus* bacteriophages display proteomic similarity to phiKZ-like bacteriophages, particularly with their structural proteins, with little genomic synteny. These results suggest the phiKZ-like bacteriophages are highly divergent, derived from a common ancestor and successful in a wide range of ecological niches. It is highly likely that *Agrican357virus* family evolved through both mutational divergence and modular evolution (acquisition of larger regions of DNA, or modules), which is a common phenomenon in bacteriophages (Botstein, 1980), and yet there is extremely low variance in all isolates thus far (>94% ANI). Such high conservation in these large genomes may reflect selective forces on a majority of the genome, which is for the most part uncharacterized. The great challenge ahead is both the abundance of bacteriophages that are completely uncharacterized, and the abundance of novel proteins harbored in their genomes.

## Data Availability

All datasets generated for this study are included in the manuscript and the Supplementary Files.

## Author Contributions

RS designed and wrote the manuscript, performed genomic and proteomic analysis, executed host range, EPS depolymerase cloning, halo zone and burst size experiments, prepared samples for electron microscopy and mass spectrometry, and drew all the figures and tables. KB, BP, and OS performed the structural prediction of proteins and contributed to writing. TN and EY helped with Dotplots. DB contributed with funding and ideas and also edited the manuscript. JG is the corresponding author and principal investigator of this study and as such contributed to the design and execution of experiments, oversaw all students, edited the manuscript, and contributed with funding. All authors read and approved the submitted version.

## Conflict of Interest Statement

JG is working on licensing a phage-based therapy for fire blight. The remaining authors declare that the research was conducted in the absence of any commercial or financial relationships that could be construed as a potential conflict of interest.

## Footnotes

1 http://cobamide2.bio.pitt.edu

2 https://cpt.tamu.edu/galaxy-pub/

3 https://galaxy.pasteur.fr
