# Supplementary material for: A Novel, Highly Related Jumbo Family of Bacteriophages That Were Isolated Against Erwinia
Source: Front Microbiol. 2019 Jul 23;10:1533. doi: 10.3389/fmicb.2019.01533 (PMC6690015; doi:10.3389/fmicb.2019.01533)
Supplement: Supplementary file 1 [file Data_Sheet_1.PDF]

Supplementary Material

Supplementary Table 1. Average Nucleotide Identity of *Agrican357viruses*- Deimos-Minion, RAY, Special G, Simmy50, Desertfox, Mortimer, Bosolaphorus, MadMel and Ea35-70 suggests a single cluster of related bacteriophages with little or no similarity to other bacteriophages. The genome sequences of all eight bacteriophages were compared against one phage from each potential cluster formed in whole genome dot plot analysis. Bacteriophages used in this analysis are *Erwinia* bacteriophages Deimos-Minion (KU886225) , RAY (KU886224), Simmy50 (KU886223), Special G (KU886222), Desertfox (MG655268), Bosolaphorus (MG6552687), MadMel (MG655269), Mortimer (MG655270), Ea35-70 (NC_023557**),** PhiEaH1 (NC_023610), Rexella (KX098390), Huxley (NC_031127), Yoloswag (KY448244), Joad (MF459647), SunLIRen (MH426725), phiEa21-4 (NC_011811) and Pavtok (MH426726) *Salmonella* phage SPN3US (NC_07402), *Pseudomonas* phage phiKZ (AF399011), 201phi2-1 (NC_010821) and *Ralstonia* phage RSL2 (AP014693).
Color coding based on decreasing order of similarity: dark grey (<100%) to light grey (<50%).

	Ea35-70	Special G	Simmy50	Deimos-Minion	RAY	Mortimer	Bosolaphorus	Desertfox	MadMel	Joad	SPN3US	Huxley	201phi2-1	Yoloswag	phiKZ	RSL2	phiEaH1	Rexella	SunLiRen	phiEa21-4	Pavtok
Ea35-70	100																				
Special G	95.3	100																			
Simmy50	95.5	96.7	100																		
Deimos-Minion	95.3	94.5	94.7	100																	
RAY	96	96.7	97.4	95.2	100																
Mortimer	95.6	96	96.6	95.3	96.9	100															
Bosolaphorus	94.8	95.7	96.2	95.9	96.8	97.1	100														
Desertfox	95.3	94.9	94.9	96.1	95.4	96.1	96.2	100													
MadMel	94.6	96.4	95.1	96.4	95.3	95.4	95.6	96.3	100												
Joad	33.4	33	33.1	33.1	33.3	32.9	33.1	33.1	32.8	100											
SPN3US	28	27.7	27.8	27.7	28	27.6	27.8	27.8	27.5	26.7	100										
Huxley	28.3	28	28	28	28.2	27.9	28	28.1	27.7	26.8	52.6	100									
201phi2-1	25.4	25.2	25.2	25.1	25.3	25.1	25.2	25.2	25	22.5	23.2	23.2	100								
Yoloswag	25.5	25.2	25.2	25.2	25.4	25.1	25.3	25.2	25	23.7	24.5	24.5	23.1	100							
phiKZ	24.4	24.2	24.2	24.2	24.4	24.2	24.2	24.2	24.1	22.5	23.2	23.1	24.8	24.4	100						
RSL2	24.4	24.1	24.1	24.1	24.3	24	24.2	24.1	23.9	23.8	24.7	25	21	24.5	21.5	100					
phiEaH1	20.7	20.5	20.5	20.5	20.7	20.5	20.6	20.6	20.4	19.6	20.2	20.3	19	20.4	35.1	19.3	100				
Rexella	15.8	15.6	15.6	15.6	15.7	15.5	15.6	15.6	15.5	12.8	10.3	10.3	7.7	8.6	7.9	9.9	7.8	100			
SunLiRen	9.8	9.7	9.8	9.7	9.8	9.7	9.8	9.8	9.7	10.2	10.2	10.2	15.4	9.4	8.9	10.4	8.5	7.7	100		
phiEa21-4	9.8	9.7	9.8	9.7	9.8	9.7	9.8	9.8	9.7	10.2	10.2	10.2	15.4	9.4	8.9	10.4	8.5	7.7	97.6	100	
Pavtok	7	6.9	6.9	6.9	7	6.9	7	7	6.9	7.1	7.3	7.4	5.7	13.6	6	8.1	6.6	6	6.1	6.1	100

Supplementary Table 2. Putative gene products predicted to encode mechanisms of replication and DNA repair.

**Protein**	**Deimos-Minion**	**Simmy50**	**RAY**	**Special G**	**Mortimer**	**Desertfox**	**Bosolaphorus**	**MadMel**
**DNA repair proteins**								
SbcC-like proteins	gp23	gp24	gp23	gp23	gp23	gp23	gp23	gp23
exodeoxyribonuclease VIII	gp67	gp67	gp64	gp67	gp66	gp64	gp65	gp66
SbcCD, D subunit	gp244	gp244	gp238	gp242	gp244	gp239	gp241	gp242
RADZ/SF2 Helicase	gp256	gp256	gp250	gp254	gp256	gp251	gp252	gp253
UvsX protein	gp155	gp153	gp150	gp152	gp154	gp150	gp151	gp151
**Replication Proteins**								
thymidine kinase	gp118	gp116	gp113	gp115	gp117	gp113	gp114	gp114
thymidylate kinase	gp317	gp316	gp311	gp315	gp318	gp313	gp314	gp314

Supplementary Figure 1. Classification of proteins demonstrates the uniqueness of *Agrican357virus* bacteriophages due to A) abundance of novel proteins found in RAY and, B) by discovering majority of proteins with predicted function as structural genes. NCBI translated BLAST (blastx) was used to find the novel proteins in the genome of the bacteriophage RAY (BLAST hit of an e-value less than 1e-04 and no hit outside of the *Agrican357virus* bacteriophages).

A.

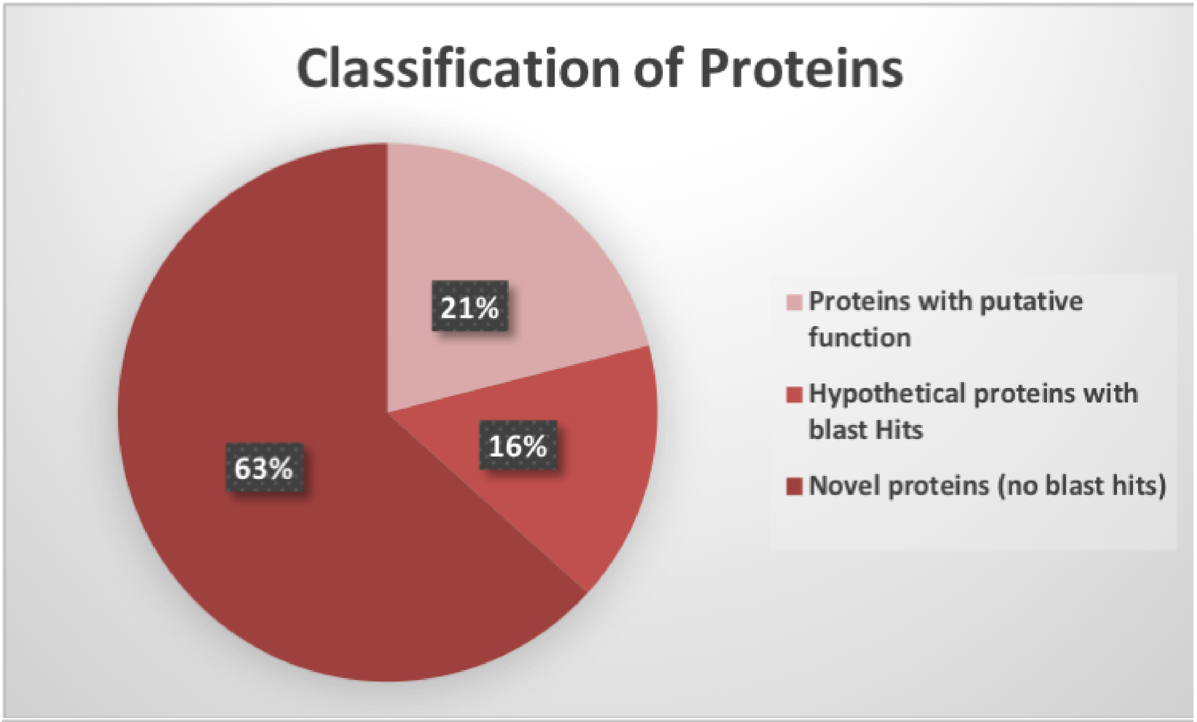

B.

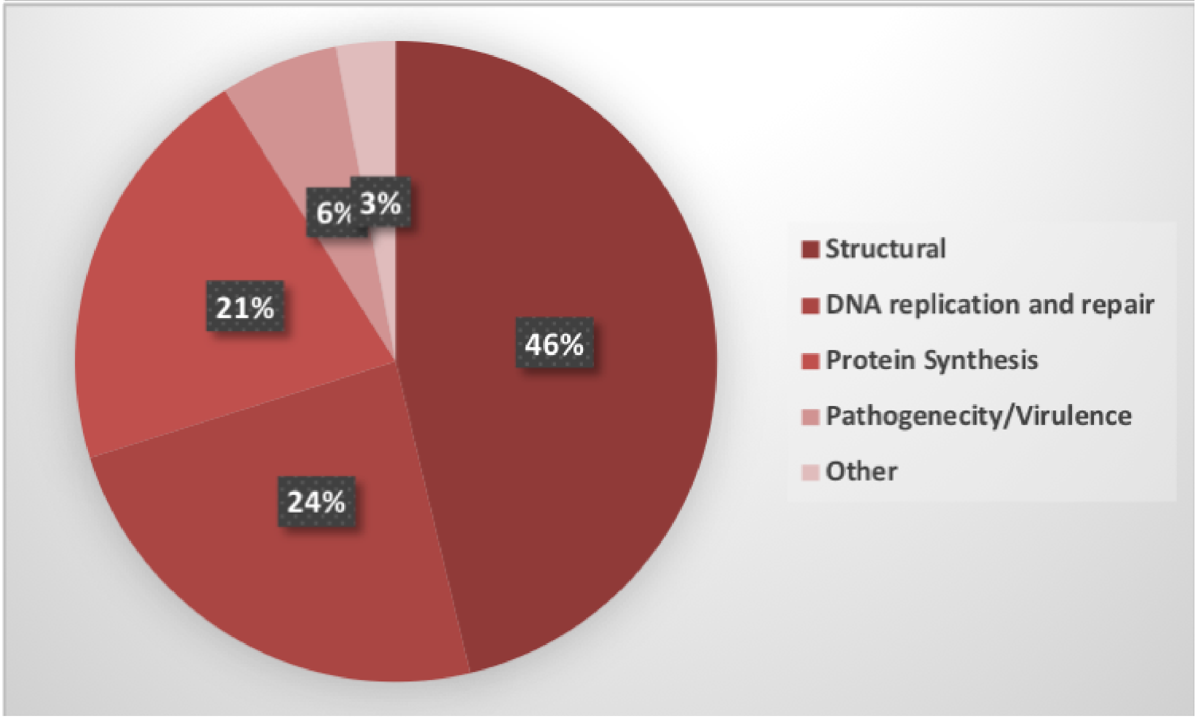

Supplementary Figure S2. Predicting putative protein structure of interesting proteins from *Agrican357virus* family via Raptor.
Conserved domains for all bacteriophages in the family were found using the NCBI Conserved Domain Database (Marchler-Bauer *et al.*, 2003; Marchler-Bauer *et al.*, 2005; Marchler-Bauer *et al.*, 2015) with the acceptable return threshold set at E-value < 3e-5. RaptorX (Wang *et al.*, 2011; Wang *et al.*, 2013; Peng and Xu, 2011) was used to predict tertiary structure and binding sites and to produce the possible images of *Agrican357virus* proteins. These predicted structures were used to show similarity between putative and known proteins as evidence that these proteins may indeed perform the given putative functions (Wang *et al.*, 2011; Wang *et al.*, 2013; Kallberg *et al.*, 2012).
The predicted fold of the proteins from the phage RAY is shown in blue and the known crystallography structures of these proteins are shown in green A) SbcCD protein of RAY with MRE11 B) Exodeoxyribonuclease VIII protein of RAY with exodeoxyribonuclease VIII of *E. coli* C) thymidine kinase of RAY with thymidine kinase from *Thermotoga maritime* D) Thymidylate kinase of RAY with thymidylate kinase of *Mycobacterium tuberculosis*. E) UvsW and F) UvsX of RAY with UvsW and UvsX from T4 help confirm putative functions.

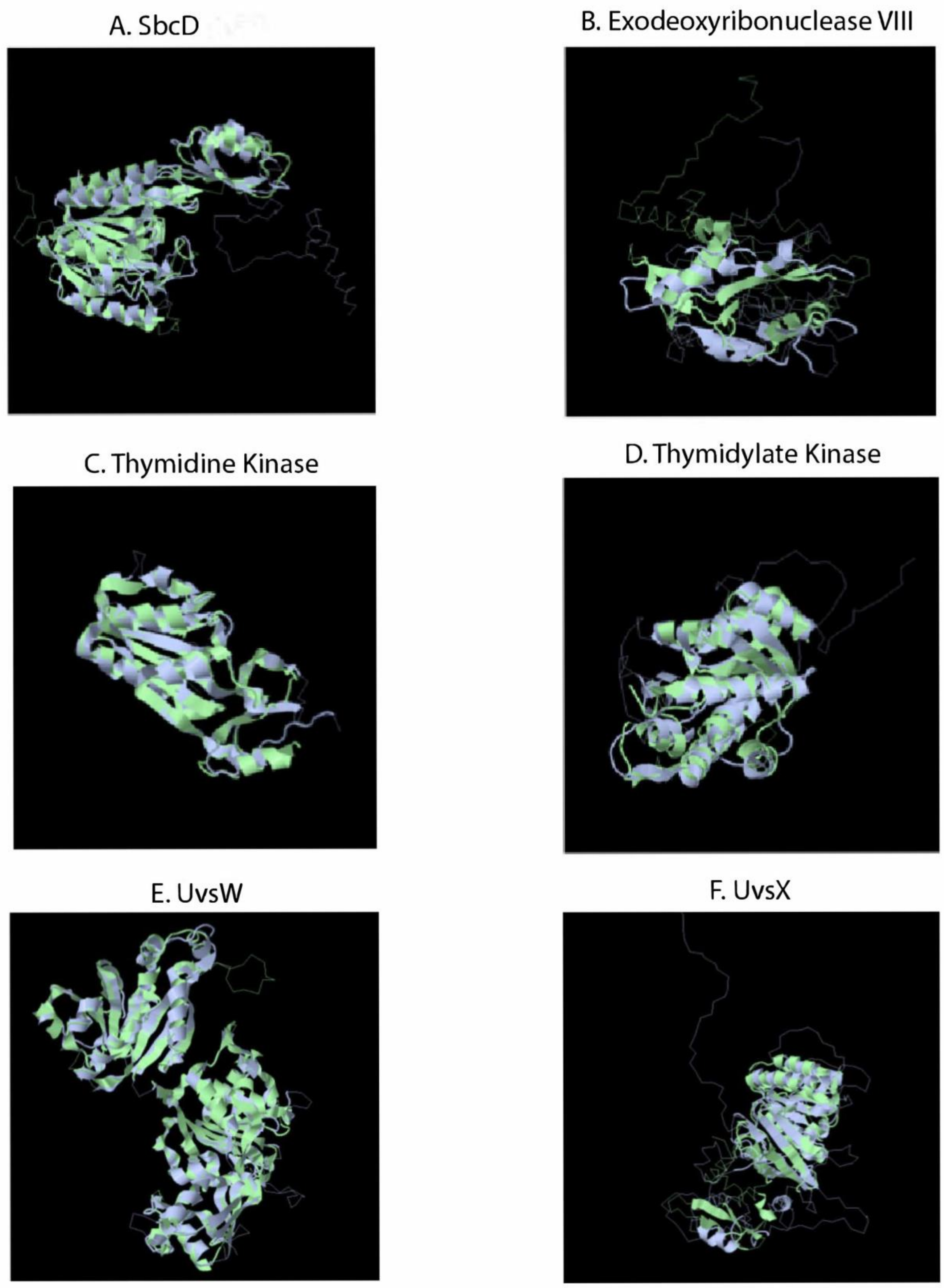
A. SbcD
B. Exodeoxyribonuclease VIII
C. Thymidine Kinase
D. Thymidylate Kinase
E. UvsW
F. UvsX
